# Lithotomy

**Published:** 1835

**Authors:** 


### XV.—Lithotomy.

This is becoming an operation of more frequent performance among the younger members of the profession, and we hail it as an omen of increased attention to the study of our science. We are the more highly gratified because, from their remote situations, in many instances, from the great seats of medical learning and surgical operations, it could hardly be presumed that they have pursued a minute course of anatomical investigation, with a view to the performance of the more important operations in surgery. But such seems to be the fact. We have recently received a short notice from Dr. Taft, of the performance of a lithotomic operation, in the Delaware county poor-house, by Ferris Jacobs, M. D. It is stated that the encrusted surface of the stone was composed of the oxalate of lime, and that the entire calculus weighed five ounces. The patient was discharged a few weeks after the operation, perfectly restored. C. R. C.

*June*, 1835.

